# Endometriosis and endometriosis-associated cancers: new insights into the molecular mechanisms of ovarian cancer development

**DOI:** 10.3332/ecancer.2018.803

**Published:** 2018-01-25

**Authors:** Amy Dawson, Marta Llauradó Fernandez, Michael Anglesio, Paul J Yong, Mark S Carey

**Affiliations:** 1 Department of Obstetrics & Gynaecology, Faculty of Medicine, University of British Columbia, Vancouver, British Columbia V6Z 2K8, Canada; 2 Department of Pathology and Laboratory Medicine, Faculty of Medicine, University of British Columbia, Vancouver, British Columbia V6T 2B5, Canada; 3 Department of Surgical Oncology, BC Cancer Agency, Vancouver, British Columbia V5Z 1G1, Canada

**Keywords:** endometriosis, ovarian cancer, molecular mechanisms, biomarkers

## Abstract

Endometriosis is a fascinating disease that we strive to better understand. Molecular techniques are shedding new light on many important aspects of this disease: from pathogenesis to the recognition of distinct disease variants like deep infiltrating endometriosis. The observation that endometriosis is a cancer precursor has now been strengthened with the knowledge that mutations that are present in endometriosis-associated cancers can be found in adjacent endometriosis lesions. Recent genomic studies, placed in context, suggest that deep infiltrating endometriosis may represent a benign neoplasm that invades locally but rarely metastasises. Further research will help elucidate distinct aberrations which result in this phenotype. With respect to identifying those patients who may be at risk of developing endometriosis-associated cancers, a combination of molecular, pathological, and inheritance markers may define a high-risk group that might benefit from risk-reducing strategies.

## Introduction

Endometriosis is a common and complex disease, affecting approximately 6–10% of women of reproductive age. The disease has a significant impact on women, as it is prevalent in greater than one-third of women with infertility and two-thirds of women with chronic pelvic pain [1]. Endometriosis is defined by the presence of ectopic endometrium (including glands and stroma) in extrauterine locations such as the rectovaginal septum, peritoneal surfaces, or ovaries [2]. The disease varies considerably in its presentation and severity. Patients experience a wide range of symptoms from asymptomatic disease to significant dyspareunia, dysmenorrhea, and infertility [3]. Interestingly, symptom severity does not necessarily correlate with the clinical extent of disease, and disease progression can be highly unpredictable. These gynaecologic disorders rarely cause mortality; however, they may have a significant impact on a patient’s quality of life, and some cases may represent risk factors for gynaecologic malignancies such as cancer.

While practicing physicians are very familiar with the clinical features of endometriosis, emerging molecular technologies are enhancing our understanding of the disease in order to improve knowledge and treatment management. The purpose of this review paper is to discuss some of the recent progress that has been made as a result of the study of the molecular biology of endometriosis. Some of the key questions relating to the etiology, progression, and malignant transformation of endometriosis are now being clarified. In this review, we provide some interesting perspectives on this emerging information.

## Risk factors and etiology of endometriosis

Significant risk factors for the development of endometriosis include conditions that increase the chances of retrograde menstruation and genetic/hereditary factors. Risk factors for endometriosis include early menarche, nulliparity, dysfunctional uterine bleeding, aberrant estrogen levels [4–7], and low body mass index [5]. Factors such as adequate exercise may be preventative against development of endometriosis [8]. It is known that the incidence of endometriosis in women with first-degree relatives who also have the disease may be up to ten times higher than that of the general population [9, 10]. There is likely to be a multifactorial genetic predisposition for endometriosis, and genome-wide association studies (GWAS) have indicated single-nucleotide polymorphism (SNP) profiles which may increase the risk of endometriosis in individuals [11]. In 2012, Nyholt *et al* [12] identified 18 genomic regions harboring 38 putative endometriosis-associated SNPs in a GWAS involving 4,604 cases of endometriosis. Among the significant aberrations identified were SNPs associated with the *WNT4 gene,* known to be critical in reproductive tract differentiation and development in mammalian females [13, 14] as well as steroidigenesis [15], *VEZT*, shown to be downregulated in gastric cancers [16], and *GREB1*, an estrogen-regulated gene shown to be important in several hormone-responsive cancers [17, 18]. Another GWAS on 2,109 cases of endometriosis in 2013 performed by Albertsen *et al* also showed that SNPs associated with WNT4 were associated with the development of endometriosis [19], confirming results previously seen by Uno *et al* in 2010 [20] and Painter *et al* in 2011 [21]. A recent GWAS meta-analysis by Uimari *et al* in 2017 indicated certain cellular control pathways which were enriched in endometriosis; MAPK-related pathways controlling cell survival, migration, division, and gene expression, as well pathways involved in extracellular matrix structure [22]. Also in 2017, Sapkota *et al* identified five novel loci in sex steroid hormone pathways associated with endometriosis risk (*FN1, CCDC170, ESR1, SYNE1* and *FSHB*) [23]. While GWAS data can provide an insight into genomic aberrations that predispose to endometriosis, further genetic and functional investigation is necessary in order to fully understand the underlying mechanisms responsible for the disease phenotype [24].

**Figure 1.**
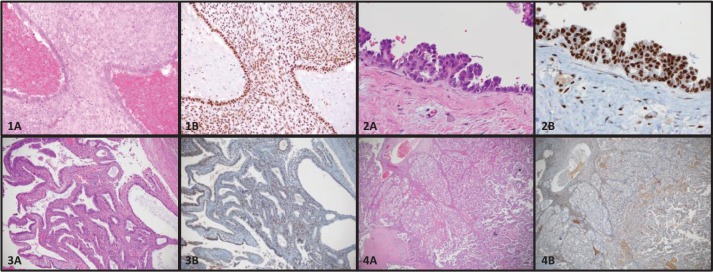
Photomicrographs of endometriosis and EAOC stained by hematoxylin and eosin (A) or immunohistochemistry for BAF250a (B). 1) Typical endometriosis lesion (1A) maintaining BAF250a expression (1B). 2) Atypical endometriosis lesion (2A) demonstrating cellular hyperplasia maintaining BAF250a expression (2B). 3) Endometrioid ovarian carcinoma (3A) with BAF250a loss (3B). 4) Clear cell ovarian carcinoma (4A) with BAF250a loss (4B).

There are several theories pertaining to the origin of endometriotic lesions. Ectopic implants of endometrial tissue may arise by retrograde menstruation (the reflux of endometrial tissue into the peritoneal cavity), resulting in the implantation and proliferation endometrial glands and stroma on extrauterine surfaces [25]. An alternate theory of coelomic metaplasia focuses on the *de novo* formation of endometrial glands and stroma by abnormal tissue differentiation from non-endometrial tissues [26]. Other common theories of origin suggest a lymphatic or haematogenous spread of endometrial tissue by dissemination through endothelial channels [27]. Based on recent molecular studies, it is interesting to speculate on the origins of endometriosis. Although there may be more than one possible explanation, current evidence supports the theory that endometriosis arises from the establishment, proliferation, and differentiation of a stem cell [28], or the implantation of endometrial cells secondary to retrograde menstruation. Stem cells can be extracted from menstrual blood and these cells show both mesenchymal and embryonic cell markers [29]. Presumably these stem cells have the capacity to give rise to both cell types (endometrial glands and stroma). Alternatively, retrograde menstruation and implantation of both endometrial glandular and stromal cells could give rise to endometriosis. Figure 1 (1A and 1B) shows an example of both glands and stroma in a typical endometriosis lesion.

In a recent study of deep infiltrating endometriosis, mutations found in glandular epithelium were not found in surrounding stroma in both of the two cases analysed [30]. This suggests that the stroma could result from metaplastic change induced by the glandular epithelium. It is of interest in the development of patient-derived xenografts that the stromal tumour component is induced and derived from the mouse tissues [31, 32]. Eutopic endometrial cells with significant changes in their transcriptomes have been reported in women with endometriosis compared to women without endometriosis, indicating abnormalities that may predispose endometrial tissue to implant in extrauterine locations [33].

Interestingly, Barrett’s oesophagus is a disease that has been extensively studied and shares a number of important features with endometriosis, including an increased risk of cancer [34]. Barrett’s oesophagus was traditionally thought to result from the metaplastic transformation of squamous epithelium. Inflammation and cell injury from acid reflux results in the formation of glandular epithelium replacing the normal stratified squamous epithelium. Evidence now suggests that the ongoing inflammation imposes selection pressure for mucin-producing cells and that these cells can better resist the acidic environment [35]. Further research by a number of investigators suggests that the cell of origin may in fact reside in the submucosal glands of the oesophagus supporting the theory that transdifferentiation (metaplastic change) of the basal squamous cells may not give rise to the columnar epithelium [36–38]. This information provides little support for the theory that endometriosis is a metaplastic change of either peritoneum or embryonic rest cells, particularly when the differentiation of a single cell must result in two different cell types [39]. Knowing that deep-infiltrating endometriosis lesions display a unique somatic mutation signature and that distinct lesions have demonstrated clonal relatedness [30], we postulate that cases of extra-peritoneal endometriosis seem even less likely to have arisen from metaplastic changes and instead are likely the result of lymphatic or haematogenous spread. Further functional analyses are required to better understand the origin and establishment of endometriosis lesions.

## Endometriosis as a cancer precursor: the historical perspective

A number of gynaecologic cancers of specific histotypes are thought to originate from endometriosis. In 1927, Sampson first published a report of a malignancy associated with endometriosis wherein he described specific criteria for endometriosis-associated ovarian cancers (EAOCs) [27]. First, there must be a clear example of endometriosis in association or close proximity to the cancer. In addition, no other primary tumour site must exist and the histology of the tumour must be consistent with an endometrial origin. Endometriosis is frequently described in association with clear cell and endometrioid ovarian cancers. A study by Vercellini *et al* in 1993 had showed a 26.3% history of endometriosis in women with endometrioid ovarian cancers (EnOC), 21.1% in clear cell ovarian cancers (CCC) [40]. The occurrence of synchronous endometriosis in ovarian cancer lesions was shown to be 40.6% in CCC, and 23.1% in EnOC in 1997 [41]. In a large Canadian database of ovarian cancers, endometriosis was identified in the final pathology reports in 51% of CCC and 43% of EnOC [42].

In 1953, RB Scott amended Sampson’s original criteria, to add an additional criterion stating that the endometriosis associated with cancers must show a morphologic progression from benign to malignant in a contiguous fashion. This transformation was further characterised by LaGrenade and Silverberg in 1988 who described what appeared to be a premalignant precursor, so-called atypical ovarian endometriosis [see Figure 1 (2A)] [43]. Atypical endometriosis [AE, see Figure 1 (2A and 2B)] may be seen relatively frequently associated with endometriosis-associated cancers. Reported rates of AE vary from 20% to 80% depending on the series [42–44]. Reasons for such variation in reported rates is that there is a lack of agreement on pathological criteria for the diagnosis of AE, and the diagnosis is uncommon, noted in only about 2–3% of endometriomas [45]. Stamp *et al* found that with more careful pathology review on archived formalin fixed paraffin-embedded tissue sections, the diagnosis of AE was made twice as frequently on pathology review compared to the final diagnosis on the original reports. Moreover, these same authors searched the pathology records of a large tertiary hospital database spanning 15 years, and only 8 cases of AE were found in ovarian endometriomas without an associated cancer [42].

While endometriosis is a common disease, the overall risk of an endometriosis-associated cancer remains low. In a large epidemiological study, the overall frequency of ovarian cancer arising in a patient with a diagnosis of endometriosis was 0.3–0.8%, a risk that was 2–3 times higher than controls [46]. Interestingly, these epidemiological studies show an association with specific histological subtypes of ovarian cancer. This information supports the historical pathological observations that clear cell ovarian and endometrioid ovarian carcinomas may arise from endometriosis. Other neoplasms such as seromucinous borderline [47, 48], low-grade serous ovarian carcinomas [49], adenosarcomas [50, 51] and endometrial stromal sarcomas [52] may also arise from endometriosis [49, 53–55]. CCC and EnOC together represent the second and third most common epithelial ovarian cancers (approximately 20% of all cases) [56] and the only subtypes wherein a direct clonal relationship between endometriosis, as a direct precursor, and the cancer has been made [54, 57]. Better understanding of the precursor lesions which lead to these cancer types will improve their prevention and diagnosis.

## Disease characteristics and clinical overview

The clinical diagnosis of endometriosis is challenging, as signs and symptoms may vary considerably and there is a lack of reliable diagnostic serum biomarkers [58]. Elevated levels of the biomarker CA-125 are not specific since they can indicate the presence of various gynaecologic pathologies, such as endometriosis, ovarian cancers or inflammation [59]. In some cases, levels of the serum biomarker HE4 can be used to distinguish endometriosis from ovarian and endometrial cancers [60]. In many patients, endometriosis is clinically suspected based on history and examination, and treated empirically with hormonal therapy (e.g., estrogen-progestin contraceptives or progestin-only therapies) without surgery [61]. A reliable diagnostic serum biomarker would represent a major advance for clinically diagnosing endometriosis [58].

Surgery with histological confirmation of ectopic endometrial glands and stroma remains [see Figure 1 (1A)] the gold standard for diagnosis [62, 63]. Surgery is generally reserved for patients who fail medical therapy, or who desire pregnancy, and is usually performed by laparoscopy [64–68]. Gonadotropin-releasing hormone agonists are also used in severe cases. Other potential treatment options include hormone receptor (estrogen or progesterone) modulators, immune modulators, aromatase inhibitors, and anti-angiogenic drugs [69–71]. There are a number of excellent clinical reviews published on endometriosis. These manuscripts offer very comprehensive discussions of the clinical features of endometriosis and its treatment, and are therefore not further discussed in this review [64, 69, 72, 73].

There are three subtypes of endometriosis described in patients that can be clinically identified: ovarian endometriosis (endometriomas), superficial peritoneal endometriosis, and deep infiltrating endometriosis. Endometriotic lesions have been shown to have altered estrogen biosynthesis and are estrogen dependent. Estrogen dysregulation appears to be linked to increased aromatase expression and activity [74]. Additionally, resistance to the anti-proliferative effects of progesterone is associated with a shift in estrogen receptor isoform expression resulting in estrogen-mediated inhibition of progesterone receptor expression [75]. Furthermore, epigenetic alterations related to alterations in hormonal signaling pathways have also been reported [76]. In addition to imbalances in hormone regulation, oxidative stress caused by high iron levels has been reported to lead to increased levels of somatic mutations [77, 78]. Vercellini’s ‘incessant menstruation hypothesis’ [79] cites retrograde transport of blood, endometrial tissue, and carcinogens as potentially leading to the genesis of both endometriosis, as well as serous, endometrioid, and clear cell ovarian cancers. High levels of oxidative stress and iron exposure are the consequence of the inflammatory response that may arise from either retrograde menstruation or the endometriosis itself. Oxidative stress leads to increased angiogenesis, endometriosis proliferation, and selective iron-mediated DNA damage leading to potential oncogene mutations [80]. Local and systemic inflammatory responses likely play a key role in the cause of chronic pain and infertility [81–85]. Thus, inflammatory responses, along with the known hormonal dysregulation in endometriotic implants, may drive carcinogenesis [86]. While some EAOCs arise with obviously associated endometriosis, this is not always the case. Interestingly, many EAOC lack identifiable endometriotic precursor lesions as they may be destroyed by the resulting EAOC or simply not detected due to sampling limitations. We suspect, based on the frequency of adhesion formation in cases of EAOC, that the incidence of pre-existing endometriosis in such cases is very high.

## Development of EAOC from endometriosis

The concept that endometriosis is the precursor lesion of some ovarian cancer subtypes has been supported by a number of lines of investigation. Initially as mentioned above, the association was noted by pathological methods, though epidemiological, and genetic studies have been valuable [25, 27, 40, 43, 44, 49, 87–92]. Jiang *et al* described some of the first studies suggesting a molecular basis linking endometriosis with cancer development in 1998. They demonstrated the same loss of heterozygosity (LOH) events in endometriosis lesions and adjacent endometrioid ovarian cancers in 82% of cases examined (*n* = 11) [87]. Similar evidence was reported by Prowse *et al* in 2006, who demonstrated common LOH events in both endometrioid and clear cell OCs and their associated endometriosis lesions, including both adjacent and contralateral endometriosis [90]. Additionally, LOH resulting in PTEN loss may be an early driver event in the genesis of in EAOC from endometriosis [93, 94]. Over the last 7 years, sequencing and immunohistochemical studies have provided confirmatory evidence that mutations found in endometriosis-associated cancers are found in adjacent endometriosis. These sequencing studies clearly demonstrate a clonal relationship between benign and malignant counterparts confirming that the cancers have fact arisen from the endometriotic lesions [42, 54, 57, 95].

Somatic mutations and other genomic aberrations are found in endometriosis that have been implicated in the development of cancer. Mutations in *TP53* [96, 97] *KRAS* [30, 98], *PTEN* [94], *PIK3CA* [99, 100], and *ARID1A* gene regions [54, 57] have been described. Loss of expression of mismatch repair enzymes [101], microsatellite instability [102], and tissue-specific gene copy-number changes [103, 104], may also be seen in endometriosis lesions. LOH in endometriosis at known oncogenic loci is also frequently seen [94, 105–110]. SNPs that are associated with oncogenic transformation (seen in GWAS datasets) have been identified in cases of endometriosis [12, 19–21].

A high degree of inflammation, like that which is found in endometriosis, is a risk factor for the development of other cancers, similar to what is seen in some cases of Barrett’s oesophagus [111]. Dysregulation of gene expression in the complement pathway has been shown in endometriosis compared to normal tissues by Surwanyashi *et al* in 2014. The same authors also demonstrated linkages between upregulation of the complement pathway and upregulation of KRAS and PTEN-regulated pathways, both frequently involved in oncogenesis and maintenance of the cancer phenotype *in vitro* [112]. The complement pathway has been linked with supporting tumour growth through various mechanisms [113]. In 2015, Edwards *et al* demonstrated that 85% of atypical endometriosis lesions demonstrated a cancer-like immunological gene signature, compared to 30% of typical endometriosis lesions [114]. In 2015, a meta-analysis reported by Lee *et al* including over 15,000 ovarian cancer patients, evaluated the 38 putative endometriosis-associated SNPs identified by Nyholt in 2012 [12]. Eight of these were associated with significant risk for ovarian cancer (rs7515106, rs7521902, rs742356, rs4858692, rs1603995, rs4241991, rs6907340, and rs10777670) [115]. Also in 2015, Lu *et al* demonstrated shared genetic risk between endometriosis and epithelial ovarian cancer, particularly clear-cell and endometrioid histotypes using genome wide association (GWAS) datasets [89].

**Figure 2.**
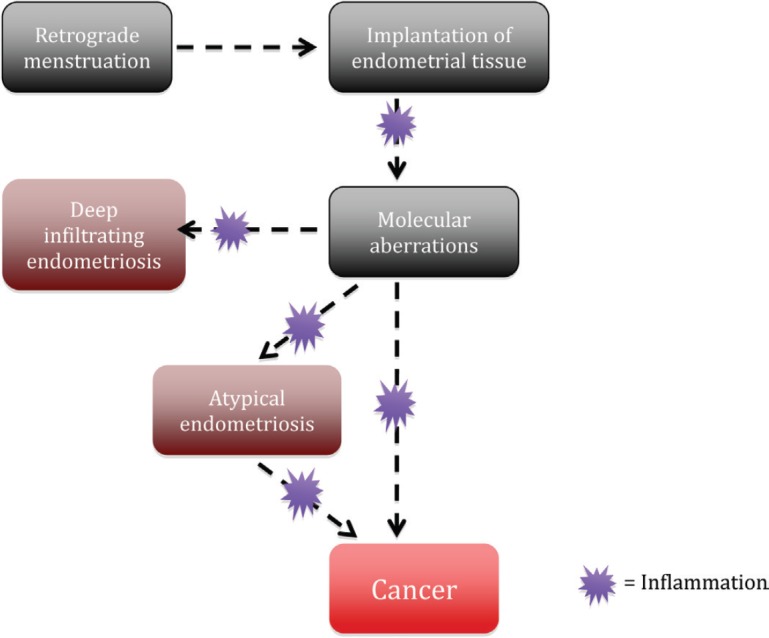
Potential process of the establishment and evolution of endometriosis lesions to EAOCs.

*ARID1A* is a tumour suppressor gene that was found to be mutated in a considerable number of EAOC [57]. Investigators were initially very excited to find that up to 42–61% of CCC and 21–33% EnOC show loss of the corresponding *ARID1A* gene protein expression (BAF250a) on IHC [see Figure 1 (3B)] [42, 57, 116]. ARID1A regulates important cellular functions (proliferation and genomic stability) as a tumour suppressor gene; therefore, it was thought that it might play a role in the transformation of endometriosis to cancer [117]. In 2015, Anglesio *et al* demonstrated that clear-cell ovarian carcinomas shared many mutations with associated concurrent endometriosis lesions, including mutations in *ARID1A*. Shared mutations in *PIK3CA* were also detected between endometriosis and clear-cell lesions, an event occurring in early progression mechanisms in other cancer types [54]. This study clearly demonstrated described mutations in contiguous endometriosis shared by EAOC, and even some distant lesions contained the same (*PIK3CA and ARID1A*) mutations. Studies examining BAF250a expression by IHC show that in just over half of the reported cases of EAOC, loss of BAF250a expression is seen the majority of the time (67–80%) in areas of contiguous endometriosis or atypical endometriosis (see Figure 1), and that a loss of Baf250a protein expression seemed to be an early molecular event in the development of Baf250a-negative EAOC [42, 95, 118]. Interestingly, *ARID1A* mutations are not sufficient on their own to cause cancer [119]. In support of this observation, Borrelli *et al* described partial loss of BAF250a in normal endometrium in the absence of cancer [120]. An important study recently reported that that 65% of cancer-causing genomic aberrations are random DNA repair abnormalities [121]. Taking this information into context, one can conclude that BAF250a loss in endometriosis could represent an EAOC precursor lesion; however, *ARID1A* mutations are neither a necessary driver mutation nor a significant determinant of the malignant phenotype. The presence of mutations in endometriosis is a sign of broader genomic disruption leading to the development of EAOC. Figure 2 shows a schematic of the establishment and evolution of endometriosis lesions to EAOC. Studies have been done comparing patient outcomes in EAOC based on the presence or absence of BAF250a expression. Based on the available evidence, it has yet to be determined as to whether there are differences in prognosis or treatment outcomes related to BAF250a loss in EAOC [122, 123]. There are few identifiable proteomic changes in a panel of proteins evaluated by reverse phase protein array (RPPA) suggesting that BAF250a loss does not define a specific proteomic signature [124]. Additionally, the presence or absence of an endometriosis precursor lesion in EAOC has not been associated with a change in overall disease outcome [125].

## Endometriosis as a neoplasm

Deep infiltrating endometriosis is an interesting rare subtype of endometriosis which was recently subjected to genomic evaluation. Deep endometriosis has a propensity to locally invade surrounding structures (bowel, bladder, ureter) but rarely metastasises. Anglesio et al demonstrated the presence of somatic mutation events in 79% of 24 cases, with 26% of all cases screened harbouring statistically significant somatic mutations in known cancer driver genes such as *KRAS*, *PIK3CA, ARID1A,* and *PPP2R1A.* In the analysis of a smaller subset of samples, mutations in *KRAS* found to be present in the epithelial component of endometriosis lesions were absent in the stroma. Additionally, one patient was found to have the same *KRAS* mutation in three spatially distinct endometriosis lesions. While these molecular events are commonly found in EAOCs, this study demonstrated their presence in deep infiltrating endometriosis. While traditionally oncogenic driver mutations (like *KRAS*) were present in a quarter of samples, they did not appear to indicate the likelihood of the lesion to progress into a gynaecologic cancer nor appear to be required for the development of the deep-infiltrating lesions. This suggests that additional or different molecular mechanisms may be at play in the development of endometriosis, and future research using a broad array of molecular technologies (epigenetic, splicing aberrations, complex chromosomal rearrangements, transcriptome, proteome and post-translational changes) to investigate the functional biology of endometriosis is warranted. Novel molecular technologies may also help explain the biology of clonally identical lesions in the same patient. Finally, the unusual presence of endometriosis in lymph nodes has been described, with some cases showing BAF250a loss [120]. Thus, one might expect that these very unusual cases are molecularly distinct as they mimic locally metastatic cancers. Perhaps even the deep-infiltrating subtype of endometriosis, which demonstrates unequivocal invasion of surrounding tissues, may be more appropriately considered a neoplasm than a benign condition. Better understanding of the molecular pathology of this disease may provide useful strategies to diagnose and treat complex cases, with the goal of reducing morbidity and disease complications like infertility.

## Prevention strategies for EAOCs

Endometriosis is highly prevalent in women of reproductive age, causing dysmenorrhea, chronic pelvic pain, infertility and in some instances even cancer. With our rapidly advancing knowledge of neoplastic diseases and modern technologies, the application of molecular science will hopefully provide us with an opportunity to identify the etiology of endometriosis, and the patients with endometriosis who are at risk of developing cancer. In order to separate patients at risk of EAOC from those who will continue to have benign disease, the application of highly sensitive and specific novel molecular biomarkers should be explored. Likely a combination of epidemiological, pathological and molecular risk stratification will be required. The finding of atypical endometriosis in the absence of cancer is rare and the risk of developing cancer in such cases is unknown. Pearce *et al* [53] showed that women with a history of endometriosis in higher-risk genetic groups had up to a 4–9% lifetime risk of developing ovarian cancer after statistical adjustment for oral contraceptive use and parity. This high-risk group represented 1.8% of the total study population of over 5,000 women with ovarian cancer. This type of risk stratification could serve to identify a baseline population for further follow-up and molecular testing.

Additional research should be directed to the discovery of biomarkers that identify cases of endometriosis with oncogenic potential, the goal being to identify premalignant lesions and then study subsequent interventions in order to reduce the incidence of EAOCs and improve patient outcomes. Various technologies may be useful in the quest for biomarker discovery. Proteomic analysis by mass spectrometry of endometrial fluid from women with and without endometriosis has been used to putatively examine differential protein signatures between normal and gynaecologic disease conditions [126–128]. Circulating tumor DNA (ctDNA) can now be detected in blood and the presence of cancer-specific mutations may prove to be clinically useful for the diagnosis of both primary and recurrent disease, determining prognosis, and predicting treatment responses [129, 130]. Thus, studying ctDNA could also play a role in the early detection of genomic dysregulation of cancer precursor lesions now known to be present in some patients with endometriosis [131]. In rare cases of atypical endometriosis not associated with cancer, biomarker research could theoretically identify those cases with true oncogenic potential. Future research efforts should also focus on establishing model systems of endometriosis (such as cell lines or xenografts). These studies could offer important insights into the risk factors, subtype-specific molecular traits, novel therapeutic testing, and the factors responsible for the development of EAOCs.

While we work with these new technologies to identify biomarkers of EAOC risk, it is important to highlight current interventions that are known to reduce the risk of ovarian cancer, including EnOC and CCC. Regular use of the oral contraceptive pill for 5 years results in a 20–30% reduction in EnOC and CCC risk [132]. Similarly, tubal ligation is at least as effective as the oral contraceptive in reducing the risk of EAOCs, showing a reduction in EnOC and CCC risk of almost 50% [133]. As the fallopian tube is the likely conduit for key factors resulting in the etiology and propagation of endometriosis, tubal occlusion is an important consideration for those women looking for permanent contraception. In these women, opportunistic salpingectomy also has the potential to reduce their risk of serous ovarian cancers and should be considered [134–137]. The latter strategy has been shown to be cost-effective [138–140].

Further research is needed to determine the role of risk-reducing bilateral salpingo-oophorectomy (BSO) to prevent EAOCs. One recent cost-effectiveness study suggests that BSO could be used in patients who have an overall lifetime risk of ovarian cancer higher than 4% [141]. In these cases, compliance with hormone replacement therapy must be high in order to mitigate the potential long-term adverse health effects of premature menopause [142].

## Conclusion

For those women who are having surgery for endometriomas close to menopause, unilateral salpingo-oophorectomy may be considered if the endometrioma cannot be completely removed by cystectomy as most cases of EAOC arise from endometriomas. Finally, understanding the molecular biology of endometriosis will be the key to better treatments for endometriosis and guide future early detection and prevention strategies to further reduce the incidence and mortality of EAOCs.

## Conflicts of interest

The authors have no conflicts of interest to declare.
